# Dissection revisited: Deciphering bodies and ancient medical texts

**DOI:** 10.1073/pnas.2416336121

**Published:** 2024-12-02

**Authors:** Andres Pelavski, Esteban Marroquin-Arroyave, Joshua Milgram, Orly Lewis

**Affiliations:** ^a^Department of Classics, Faculty of Humanities. The Hebrew University of Jerusalem, Mount Scopus, Jerusalem 9190501, Israel; ^b^Department of Anaesthesia, Vall d’Hebron University Hospital, Barcelona 08035, Spain; ^c^Faculty of Agriculture Food & Environment, Koret School of Veterinary Medicine, Hebrew University Jerusalem, Rehovot 7610001, Israel

**Keywords:** anatomy, dissection, peritoneum, ancient medicine, Galen

## Abstract

Unlike traditional approaches to Greco-Roman medicine, which are strongly based on textual evidence, we addressed some problems, currently debated by historians, through a technique borrowed from the biological sciences: We reenacted an ancient anatomical dissection of the abdominal wall, the peritoneal cavity, and its organs (as described by Galen during the 2nd century CE, in his major treatise Anatomical Proceedings). Our aim was to clarify incongruencies about the text itself—considering the errors generated during its 14-century-long hand-copied manuscript tradition—and infer answers to contextual questions, such as the aim of the author, his audience, and the setting where the dissections took place. This hands-on practical method provided extratextual evidence to ongoing scholarly debates, which until now were mainly approached through textual scrutiny.

## Significance

This paper introduces a multidisciplinary approach to the study of medical humanities. By combining the efforts of experts in veterinary and human medicine, computer and data science, ancient history, and classical philology, our research provides a tool to address several ongoing scholarly debates. Indeed, we were able to yield alternative answers to questions that had been so far approached mainly by reading the works of ancient medical writers. In this way, this method based on real-life, hands-on experience avoids some of the caveats that extant texts from antiquity normally present, in terms of manuscript transmission and interpretation.

Scientific dissection has been performed since antiquity to deepen knowledge of the body (1), as an invaluable didactic tool (2), and more recently to improve surgical techniques (3) and optimize perioperative analgesia (4). This paper presents an unconventional application of this method to the study of the Classical humanities. Through a modern reenactment of a dissection described during the 2nd century CE by Galen of Pergamon—the great doctor of the Roman Empire, whose writings shaped the history of occidental medicine—we attempted to shed light on relevant aspects of his work and environment.

**Fig. 1.**
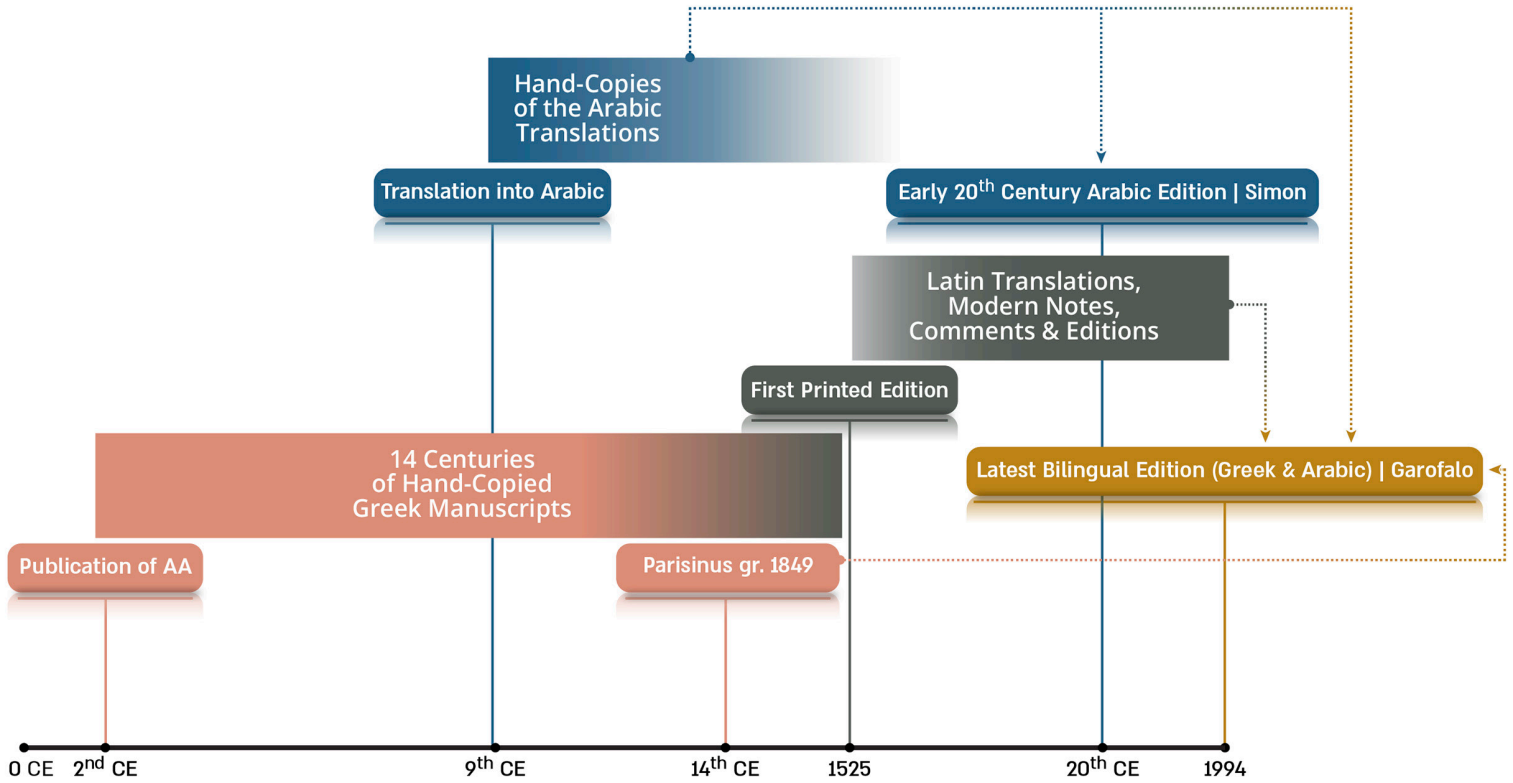
Textual problems. This figure summarizes the manuscript tradition to illustrate the scope of textual problems: After its publication during the 2nd century CE, the Anatomical Procedures (*AA*) was preserved in the Byzantine empire through successive hand-copied manuscripts (5). Its modern history began in the Renaissance, when it was first printed by the Aldine press (1525). Naturally, this technological development allowed a wider diffusion of the work, and it was subsequently translated into Latin, commented upon, and edited by humanists and later intellectuals. Nevertheless, despite the numerous versions of the text (after fourteen centuries of hand-copying with consequent scribal errors) (6), all our extant versions derive from a single manuscript—*Parisinus gr*. 1849, from the 14th century CE—which lacks a third of the work and has important *lacunae*, corrupted passages, and abbreviations difficult to interpret in the extant parts. A parallel development occurred in the Arab world (7): Around the 9th century CE, the work was translated by Hunain Ibn Isahq’s school into Arabic (in a version that was probably influenced by the translation of the text into Syriac) (8). A few extant manuscripts from this tradition preserve the entire work, from which at the beginning of the last century Simon published an edition of the parts extant only in the Arabic translation. The latest Greco-Arabic bilingual edition by Garofalo covers the parts preserved in both languages; it is mainly based on the *Parisinus* manuscript and he took into account some amendments and conjectures made by editors since the Renaissance, as well as the Arabic extant manuscripts (9).

Current understanding of ancient science relies heavily on textual sources. Accordingly, historians base most of their ideas concerning Galen and his social and cultural milieu on the author’s vast extant textual production as well as on other ancient sources with information relevant to various Galenic topics. However, these written sources are not devoid of problems. First of all, most available texts are the result of a centuries-long process of copying and recopying manuscripts, where each new transcription replicated the various human errors of its previous versions while also providing an opportunity for new copying errors. Consequently, there are several variants of most ancient texts attested by the surviving manuscripts. This makes it difficult to know what the author actually wrote, particularly in passages where the complexity of the syntax or the corruption of the handwritten codex yielded multiple variants and conjectures (Fig. 1).

**Fig. 2.**
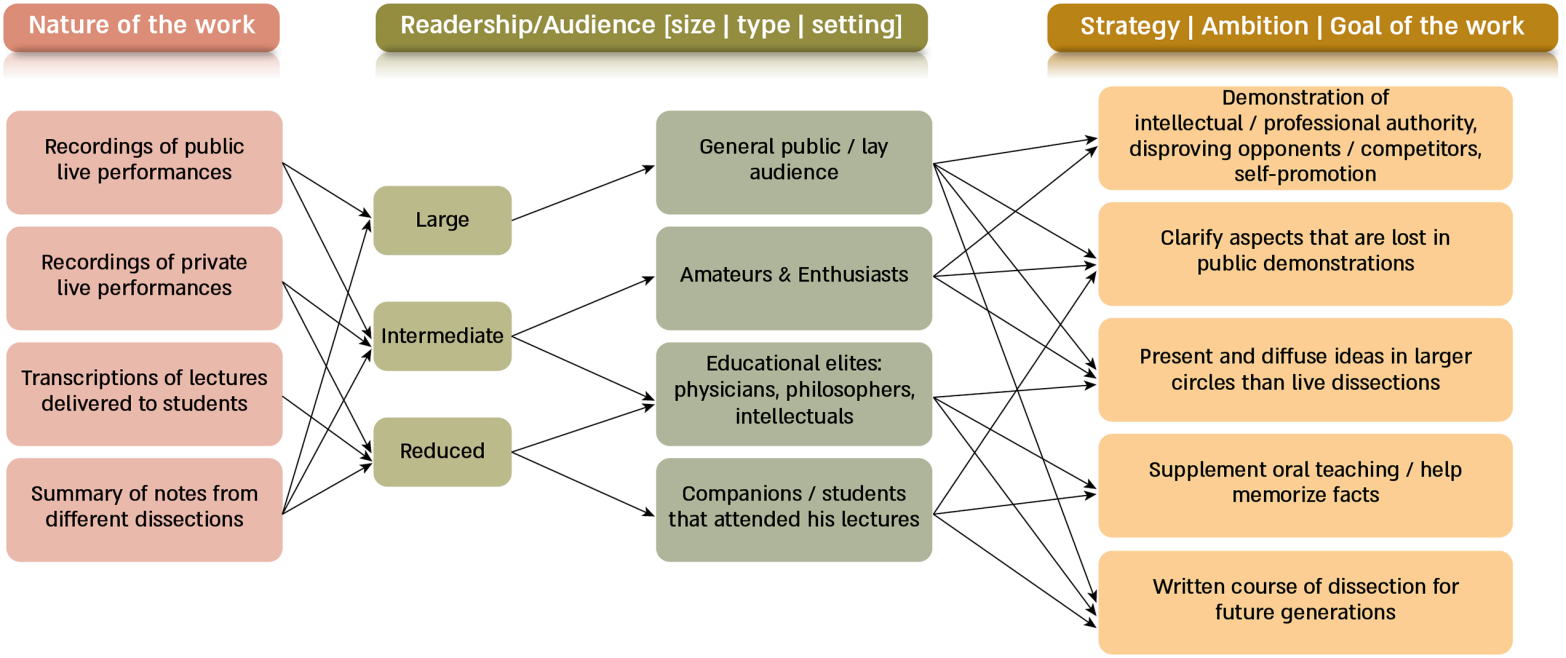
Contextual debates. Fig. 2 summarizes the debates that Galenic texts have generated among scholars during the last few decades, along with the provisional explanations that they have found within the texts themselves or among other ancient sources. Because during the 2nd century CE dissections and vivisections became frequent public performances—during which doctors theatrically exhibited their prowess while antagonizing competitors (15)—the Anatomical Procedures raises numerous questions concerning its possible nature: Did it record such live performances? Was it instead a collection of lecture notes? Or rather a summary of different dissections (whether private or public)? Naturally, the answer to these questions conditions the kind of readership or audience that it was addressed to. If the work reproduced public spectacles, the audience could have been large, composed of both lay and educated people, and the work possibly aimed at self-promotion. If, on the contrary, the book was the transcription of a lecture, the readership must have been smaller, restricted to well-educated individuals, and the goal would have likely been to supplement the teachings. As shown by the multiple and confusing arrows, various combinations of categories (nature, audience, and goals of the text) have been suggested by different scholars (12, 16–18), because, judging by the data that can be deduced from the extant textual evidence alone, no choice is conclusive.

Additionally, there are several contextual aspects that are key to understanding ancient texts—such as their audience, their goal, and their nature. These features characterize the society where the works were produced and raise heated debates among scholars, which are difficult to resolve through textual analysis alone (Fig. 2). In this regard, other disciplines have demonstrated the usefulness of reproducing historical experiments and reworking forgotten practices (10). With such experimental approaches, researchers were able to better understand their sources in various areas including archeology (11), physics (12), and alchemy (13)–to name but a few. Similarly, within the history of medicine, modern reenactments with didactic purposes have brought to life some dissections and vivisections performed by Harvey during the 15th century (14).

Our approach engages with Galen’s practical treatise (16) Anatomical Procedures (*AA*) to address several of these scholarly conundrums (9). In this massive text composed of 15 books—which was used as a key dissection guide until the Renaissance (17)—the author explains how to dissect every part of the body. As a case study, we chose the chapters on the peritoneum because it addresses the abdominal organs and their blood supply, which—beyond their anatomical characterization—are also relevant for several theoretical ideas about the functioning of the body more generally. In other words, through hands-on, step-by-step dissection of a pig’s abdominal wall, peritoneal cavity, and organs in the manner described by Galen (18), we aimed to provide answers based on actual experience to both textual and contextual debates. In this way, we attempted to reduce some of the uncertainties that arise from deductions that are based solely on transmitted textual documents. Additionally, given that this work is a practical manual in which Galen instructs his readership in how to dissect animals, we had to face the extra challenge of applicability; namely, the difficulty of translating words into action (16).

## Materials and Methods

Approval was obtained from the Ethical Committee of the European Research Council. The consent included the reenactment of five dissections on pigs according to the ancient method. We only used animals that had been previously killed for humane experimental reasons (i.e., no specimen was put down for the purpose of this experiment).

We reenacted the dissection of the abdominal wall, peritoneum, peritoneal cavity, and abdominal organs as described by Galen in *AA* books V.7 and VI.4 to 7 (pp. 311 to 320 and 357 to 381) (9). The different stages of the research were carried out by a multidisciplinary team, composed of a senior veterinary surgeon, a medical doctor, a biologist, 6 classicists, 2 Arabists, and 2 experts in 3-D modeling and data science.

### Research Process and Protocol.

Prior to the dissection, we read the relevant Galenic passages closely in ancient Greek in order to discuss their textual, lexical, grammatical, and interpretative hurdles. Although there has been some study of Galen’s anatomical ideas, and to a lesser extent his methods of dissection, the procedure—as such—has not been systematically attempted since the Renaissance at the latest (17); therefore, specific literature or any scientific input on how to perform the task is nonexistent.

Throughout this preparatory phase, we focused especially on distilling the specific instructions that Galen provides, which tend to be neither as clear nor as detailed as one would expect. We discussed different points of view among classicists and biologists until we reached a consensus regarding each point based on our interpretation of the text and the actual technical feasibility. We introduced the technical terms to our multilayer online database for ancient medical vocabulary and then translated the text into English (according to our interpretation). Finally, we compiled the dissection manual as a protocol to guide the procedure.

This manual turned the ancient document into a detailed list of consecutive actions to be performed along with the corresponding findings expected at each stage, and it also prescribed means of documentation (video, photo, and verbal). We also incorporated some remaining unanswered questions that we were expecting to clarify through the dissection into the manual/protocol. This document guided our work during the procedure, indicating which action should be performed at any given moment and how it should be documented. Our translation and the latest ancient Greek edition of the Galenic work (9) were at hand as well, and we revisited them whenever the procedure generated some doubt, or the findings did not agree with the protocol.

### Setting.

The reenactment was carried out in a contemporary anatomical laboratory at the Koret School of Veterinary Medicine of the Hebrew University of Jerusalem. The entire process was documented for later analysis with photographs and video footage. We used a video camera that zoomed in to take short clips of each action instructed by the protocol, and we took photographs of every expected finding (whether or not it agreed with Galen’s description). Additionally, there was 3-D scanning of certain structures and two fixed, continuous video cameras that recorded the whole procedure from different angles. Audio was recorded with the answers that emerged during the procedure to the questions that we had included in the manual.

### Reenactment.

The specialist veterinary surgeon—an expert in veterinary anatomy—performed the procedure according to the protocol on a female adult pig (approx. 50 kg). Additionally, we dissected the stomach and intestines of a second pig to verify certain findings. Although Galen was not explicit about the species of animal he used for this dissection, we chose a pig because he had used them for several of his other dissections and vivisections (18). The text is very seldom explicit about the instruments utilized, so we can only know from allusions in different passages that Galen used scalpels of different sizes and hooks. We decided to use modern scalpels (n. 24), forceps, and scissors.

### Postdissection Processing.

When the procedure was completed, we gathered all the images and footage and compared them with the dissection manual. This allowed us to assess the extent to which the findings corresponded with Galen’s descriptions, which of the previously formulated questions could be answered, and how accurately the instructions could be followed. Additionally, our expert in data science and digital technology built a multidimensional online digital platform with all of these findings: https://dissections.atlomy.com/ (19). This website –built on a collaborative work platform with a relational database structure (coda.io)– makes the raw and annotated data as well as our interpretations available for potential readers, thereby facilitating their engagement with the complexities of the text and the procedure described in it. Moreover, the platform offers essential visual evidence from the body referred to in the ancient text, which is generally inaccessible to most of its modern readers.

## Results

According to our predesigned protocol, the reenactment of the dissection consisted of 95 steps (which are built as a sequential dissection guide in our website). Of them, 66 (69.5%) could be satisfactorily executed or visualized, 20 (21.5%) were partially completed, and 9 (9.5%) remained unachieved. Any lack of fulfillment was in most cases due to the fact that we could either not find an element that Galen had described, or we could not see it exactly as he had described it. On other occasions, the problem was the technical difficulty in performing a certain action in the way that Galen had indicated.

### Textual Findings.

**Fig. 3.**
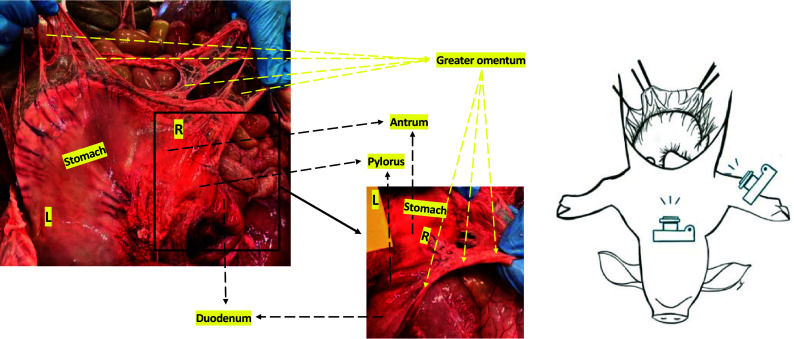
Greater omentum and duodenum. These pictures present the stomach, the duodenum, and the greater omentum from different angles. The larger image on the *Left* shows the structures as seen from the head of the pig: The stomach can be seen completely with the epiploon, which is attached to its greater curvature and—to the *Right* (R) of the stomach—some attachments between the epiploon and the duodenum are evident. The smaller image on the *Right* shows the same structures but seen from the *Right* side; from this angle, we can only see part of the gastric antrum, the pylorus and its prolongation into the duodenum, as well as the greater omentum attached to all these structures. Bearing in mind that Galen never identified the duodenum by name but considered it an “outgrowth” of the stomach, these photographs support the manuscript tradition against modern emendations. It shows how the epiploon is attached to the greater curvature of the stomach and the beginning of the duodenum, thereby suggesting that the manuscript was correct when it stated that it is attached to the *Right* component (*kôlon*) of the stomach and not to the *Right* colon (*kolon*).

There are two kinds of textual problems that the reenactment enabled us to address. First, it provided evidence that allowed us to choose between alternative variants within the manuscript tradition. As an example, different manuscripts diverge in a number of passages regarding the term *kôlon* (κώλον, with a long omega), and *kolon* (κόλον, with a short omicron). This common type of scribal error is important to resolve due to the anatomical implications here: The latter (*kolon*), which was the choice of most modern editors and translators for these passages, indicates the large intestine (what we today designate as the “colon”). However, most manuscripts contain the other option, *kôlon*, used to designate “a member, a part, a component” of something. The reenactment suggested that the manuscript tradition was correct and the editors’ amendment wrong: Chapter VI.5 of *AA* (9) explains that the epiploon—or greater omentum—has attachments to the right part of the *kolon/kôlon*. In the dissection, we could see that attachments were present between the omentum and the beginning of the duodenum but not the ascending colon. It should be noted that Galen never identified the duodenum by a specific name but instead described it as an “outgrowth” or an additional “component” of the stomach (Fig. 3).

Additionally, the reenactment clarified the exact meaning of certain subjective vocabulary, such as shades of colors or particular textures, as well as the unconventional use of certain terms. Such is the case of *sôma* (σώμα), which is not always used for its primary meaning, “body,” nor for its more philosophical/epicurean signification, “flesh” (20). The dissection showed that for Galen, the plural form of this term could have a technical but nonspecific connotation; he used it to allude generically to anatomical structures that he encountered but that were not the main object of his explanation. They could be described in more detail later (or not), but when he used the term “bodies” (or *sômata*), he intended to direct his readers to focus on something else.

### Contextual Findings.

Regarding the contextual aspects, the reenactment yielded information about the duration of the process, its technical difficulty, the facilities needed for carrying it out (such as lighting, water, and ventilation), and the proximity required for visualizing certain structures. Ultimately, all this information offers insights into the nature of the event described, the venue where it could have taken place, the kind of audience/readership that it was addressed to, and possible goals that the author aimed to achieve when writing it.

**Fig. 4.**
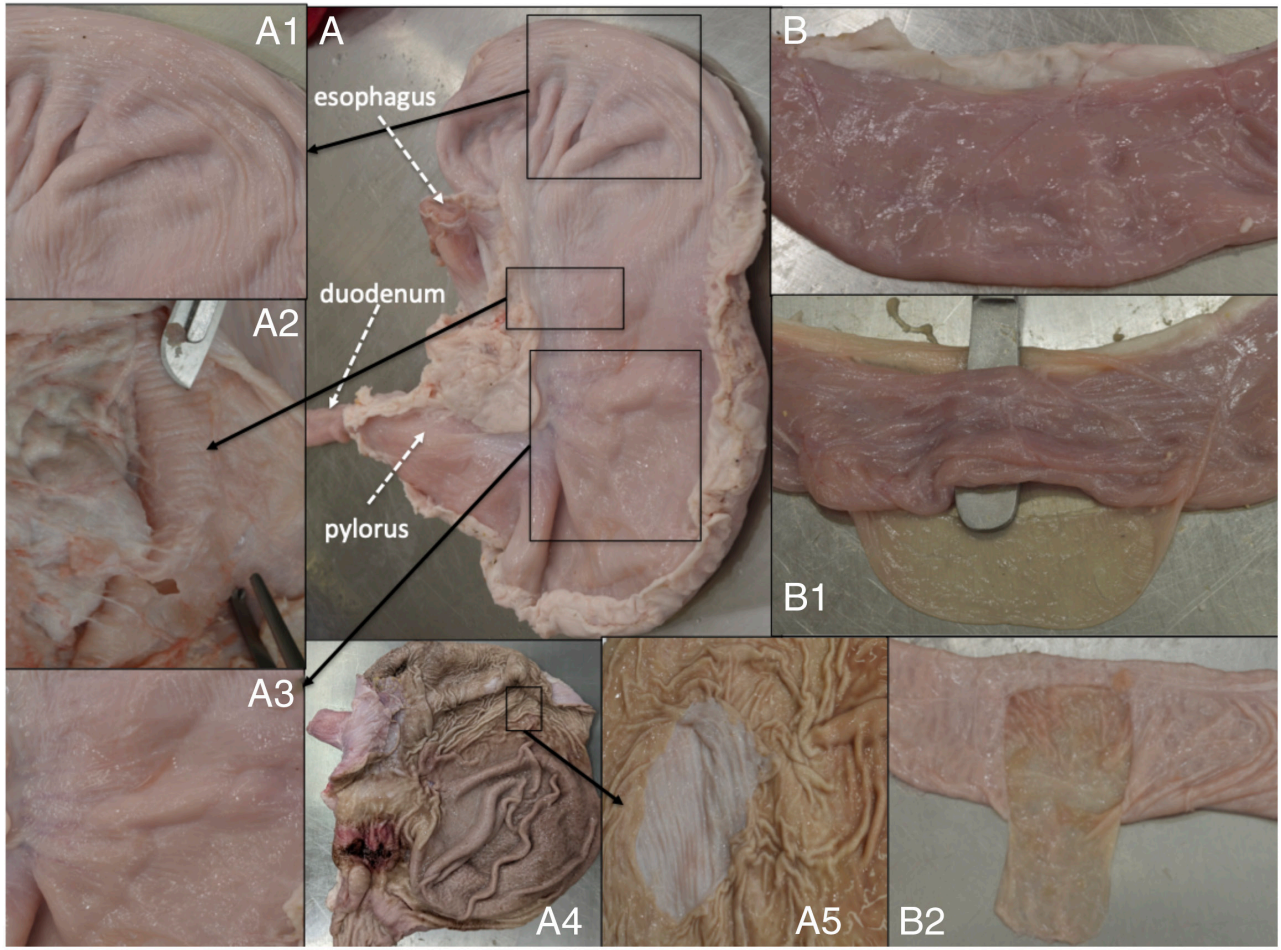
Fibers in the stomach and the intestines. (*A*) Shows an isolated stomach once removed from the abdomen. (*A*1 and *A*3) are close-up details of the external gastric wall covered by the serosa (visceral peritoneum), through which we can visualize small grooves with different directions. In A1 some grooves are parallel to the natural contour of the stomach and some are perpendicular, whereas in A3 most are perpendicular. After removing the serosa (*A*2), perpendicular grooves can also be seen. A4 Shows the stomach opened to reveal its internal (mucosal) surface with the folds of the mucosa, *rugae*. In A5 a portion of the mucosa has been removed to highlight the contrasting directions of the *rugae* and the underlying grooves of the muscularis layer. (*B*) Shows a segment of the small intestine. After separating its serosa (*B*1), some longitudinal grooves can be intuited. (*B*2) shows the circumferential *plicae* or folds of the intestinal mucosa. In current histological terms, the gastric *rugae* (*A*4) and the circumferential *plicae* of the intestines (*B*2) correspond to the folds of the inner mucosal surface of these organs. The grooves seen in the outside wall (*A*1–*A*3 and *B*1), or after removing the mucosa from inside the stomach (*A*5), belong to different layers of smooth muscle within the histological structure of the digestive tube. These layers of smooth muscle enable the movements of the organs.

The duration of the procedure exceeded 6.5 h. Some of the steps were particularly laborious and time consuming (although Galen mentioned them only in passing). A good example thereof is the dissection of the visceral peritoneum. Galen claims to have separated the visceral peritoneum from all the abdominal organs, which is technically difficult to achieve in the pig even with our modern tools (21). We could only partially remove the peritoneal layer in limited places of only some abdominal viscera. Furthermore, in those regions where we were able to remove the peritoneal layer, we could not always see what Galen describes: Chapter VI.7 of *AA* (9) explains that the stomach and the intestines present two layers of fibers. In the former, there is a longitudinal internal layer and a circular external one, whereas in the latter, the direction of the fibers is inverse. After cutting these organs open we could easily visualize, in their inner surface, the more or less longitudinal direction of the gastric *rugae* and the circular disposition of the intestinal *plicae* and colonic *haustra*; however, it was not possible for us to see any external pattern in either organ in the first specimen (no evidence can be seen in the external gastric walls shown in Fig. 3). When we repeated the procedure in the same organs of another specimen we were able to visualize the external walls of these organs covered in grooves with various directions. However, none of them was as clear and schematic as Galen claims to have seen them (Fig. 4).

In terms of environmental settings, the reenactment demonstrated that most of the structures can only be seen from a short distance—so much so that even the fixed cameras placed next to the dissecting table could not register certain steps of the procedure. For the deeper organs, it was necessary to lean forward in order to take a closer look (and zoom in with the mobile camera). Similarly, little can be distinguished if the specimen is not well illuminated.

As far as facilities are concerned, it seems impossible to perform the dissection without an abundant supply of water. The hollow viscera in particular required intense rinsing in order to allow the thorough inspection that Galen recommends. It is unlikely that Galen used an enclosed venue without good ventilation, considering the intense insalubrious odors emanating from the bowel contents and the bodily fluids of the specimen, which surprisingly Galen did not mention in his description (although he has often alluded to them in other cases).

Finally, from a modern anatomical perspective, there are other notable omissions from Galen’s text, such as the biliary tract, the pancreas, and the portal vein, to name but a few. There is no mention of any of these structures in these chapters.

## Discussion

This paper introduces the application of dissection—a technique widely used in contemporary surgery and anatomy—to the study of ancient medical texts. By reenacting a procedure carried out by Galen during the 2nd century CE, we were able to draw conclusions about his work and environment that are less dependent on the written word. As with other experimental approaches in the history of science (10), the advantage of our method is that it allowed us to provide extratextual evidence to the ongoing scholarly discussions.

### Audience and Nature of the Work.

In general terms, we were able to complete a high percentage of Galen’s instructions. However, at certain stages, decisions needed to be made on the spot. For instance, the indication “to cut the transversus abdominis muscle close to the navel” required a decision as to what “close” actually meant, i.e., where exactly the incision should be performed. Naturally, in cases like this where Galen’s writing offered no specific indication, we had to base our decision on our current anatomical understanding, and this kind of vagueness suggests that Galen considered these details either irrelevant or self-evident for his audience. Based on the level of technicality required for the procedure, we are inclined to think that he took for granted some previous knowledge. Whether Galen’s intended readers were physicians, students who had seen him do it before, or simply amateurs with some basic anatomical instruction (22), the assumption of prior knowledge supports the idea that he was not writing for a lay readership. In this way, the experiment supports—with certain reservations—some scholars’ hypothesis (22).

On the other hand, the tasks that we were not able to carry out highlight Galen’s dexterity while questioning our own (it should be emphasized that our dissection was performed by an experienced veterinary surgeon, utilizing tools that had been designed—presumably—with more advanced technology). Be that as it may, this again suggests that the author was expecting a seasoned readership. In other words, his insistence on the need for persistent practice, his repeated warnings about possible mistakes (including his own), and his flagging of certain hurdles—which could point toward a dissection guide for the inexperienced (22)—do not detract from the fact that following his instructions required substantial expertise. Furthermore, these features also speak about the nature of the work. It can hardly be thought of as the transcription of a single lecture or the written description of a live dissection (17). On the contrary, it seems as though Galen compiled in this book his accumulated knowledge and experience, thereby preserving his achievements for future generations.

### Anatomical Findings Subordinated to Physiological Understanding.

Some elements mentioned in the text that we could not see exactly as Galen describes them (despite being able to follow the instructions) pose important interpretative challenges. We considered that the more or less longitudinal gastric *rugae* and the circular *plicae* of the intestines could correspond to the internal fibers mentioned in the text. Regarding the external “tunic” of fibers in both organs, we think that the author might be referring to the grooves that we could see in the second specimen (which correspond to different layers of smooth muscle within the histological composition of those organs). Nevertheless, the direction of the fibers was not as defined and clear as described in the text. Therefore, we suspect that Galen’s observations were sometimes heavily influenced by his physiological understanding. The description of the direction of the fibers fits perfectly with his ideas about the movement of the digestive tract (23); however, the empirical proof is not as clear as he claims (Fig. 4). In terms of the difference we found in the observations between the first and the second specimen, we can speculate that the latter happened to be an empty, contracted stomach with thicker walls than the dilated, thin-walled organ we encountered in our first dissection. Other hypotheses could be that the *rigor mortis* played a part in the appearance of the second organs. Either way, given that neither the mucosal folds (the *rugae* and *plicae*) nor the layers of smooth muscle had such a clear direction in their fibers, strictly based on our evidence, we can only wonder whether in certain instances, Galen privileged theoretical views over empirical findings (or put more bluntly, if abstract speculation influenced his perception of anatomical evidence). There is yet another possibility worth exploring in future endeavors: Perhaps the fibers of other species that Galen often dissected (such as monkeys and oxen) are more consistent with his description.

### Dissection as a Powerful Philological Tool.

The data that we gathered can provide us with a powerful tool to resolve textual issues arising from different versions of the manuscript, as it provides the strongest level of evidence in favor of one version over another. Naturally, it is easier to choose between two divergent manuscripts when what is seen in the reenactment only agrees with the description in one of them. Similarly, subjective perceptions become more objective once a color or a texture is actually experienced by the reader (some authors define this as “sensual information”) (10).

### Setting and Venues of Ancient Dissections.

For the contextual debates, conversely, the dissection can provide an extra element to support or reject specific theories, but the evidence is never conclusive. Accordingly, based on our experience we could challenge the idea that the dissection of the peritoneum was one of Galen’s spectacular live performances (17). Unlike when ecstatic audiences marveled as a screaming pig became dumb because its recurrent nerve was cut, the peritoneum seems of little interest for a large crowd (24, 25). Not only are the findings less impressive, but one also needs to be close-by to observe them. Hence, if an audience was present, it was likely small. Similarly, the amount of light required to observe the details, as well as the fluids and odors emanating from the animal and its entrails suggest that the public baths (25) were not the ideal venue for this particular dissection, whereas a library or a temple (6) could only be an option provided that it was performed in an open hall or a luminous, well-ventilated auditorium within such complexes. Either way, all these assumptions remain hypothetical, and their level of certainty is not much higher than the deductions of more traditional scholarship based purely on textual evidence.

However, the need for abundant water, which our study revealed, should be particularly emphasized. Despite the perennial interest in and abundant bibliography on aqueducts and water supply in Ancient Rome (26), scholars have not included this variable when debating possible venues for dissections (6, 22–25). Consequently, some of their conclusions should perhaps be reassessed in the light of these findings.

### Omissions and Bodily Organization.

Finally, omissions are particularly relevant because the unmentioned structures were not unknown to Galen. In fact, he deliberately chose to discuss them elsewhere in this work (as well as in other treatises) but not during the dissection of the peritoneum. From a modern perspective, it seems counterintuitive that he devoted so much detail to the gastroepiploic and splenic vessels but did not mention the portal vein, or that he discussed the liver and the intestines without allusions to the biliary tract. This silence perhaps illuminates a different understanding of the organization of the body (or at least a different understanding of the way in which its study should be structured). It is worth exploring in future endeavors how Galen partitioned the study of the abdominal organs and what implications that partition had in his theoretical ideas about the interactions among such bodily parts and the workings of the body more generally.

### Limitations and Strengths.

Inevitably, our study presents certain limitations. No matter how closely we might attempt to replicate the conditions of Galen’s dissection, we can never be sure exactly how it was carried out (10). We are uncertain about the specific animal that he was dissecting or its size, and we know that unlike Galen (who either drowned or exsanguinated his specimens) (27), the pig was killed according to the Standard Operative Procedures for ethical animal handling. Additionally, our specimen was kept in refrigeration for 2 d before the procedure, whereas Galen used to start immediately after the animal died. Another limitation concerns the instruments. We are uncertain as to the tools that he used specifically for this dissection (he only mentions a blunt and a sharp scalpel and a fishhook). In other words, we can only follow his explicit instructions and try to understand and recreate some of the cultural and environmental conditions omitted in the texts (10). In any case, there are still uncertainties about the procedure that need to be acknowledged because they might have impacted the outcomes.

On the other hand, our method also has important strengths. The dissection manual–i.e., our experiment protocol–allowed for a scientifically transparent illustration of how the text was interpreted. Because we transformed every detail mentioned by Galen into a specific instruction, there is no room for doubt on how we understood the ancient guide. This, along with the visual footage, allows for the scientific reproducibility of the procedure, thereby encouraging an objective and critical analysis of our findings. Moreover, the digital platform, which follows the manual and integrates textual information, tabular data, images, video, and 3-dimensional interactive documentation, offers an innovative solution to longstanding challenges in anatomy education (28, 29). Indeed, by providing a virtual dissection experience, it aligns with principles of sustainability and leverages the educational effectiveness of advanced visualizations (30). In other words, the website optimizes its digital footprint by delivering high-quality educational content in a single, cloud-based environment, and offers a streamlined and efficient approach to accessing and interacting with complex anatomical information. This technological foundation not only supports the website’s educational goals but also aligns with modern principles of sustainable web design and digital resource management.

## Conclusions

To conclude, the reenactment of an ancient anatomical dissection of the abdominal wall, peritoneal cavity, and organs has been shown to be a valid method to approach this massive work by Galen, and it strongly supports pursuing this method in the future with other parts of the body (i.e., other chapters in the book) and other animals. Even if the experiment did not yield an indisputable level of scientific evidence, it did contribute valuable information to several current scholarly debates concerning the text, its author, and his envisioned audience. It illustrated what Galen could have seen, the way in which he engaged with what he saw, and the extent to which his findings might have been influenced by his preconceived theoretical ideas. More importantly, it illuminated aspects that normally go unnoticed in traditional scholarship where the analysis is exclusively limited to textual evidence.

## Data Availability

Multi dimentional digital platform data have been deposited in Atlomy dissection (https://dissections.atlomy.com/) (19).
